# Ocular oncology service during the COVID-19 outbreak: uveal melanoma characteristics presenting in 2019 compared to 2020

**DOI:** 10.1007/s11845-023-03310-z

**Published:** 2023-02-18

**Authors:** Aisling Mc Glacken-Byrne, Patrick Murtagh, Valerie O’Neill, Noel Horgan

**Affiliations:** Department of Ophthalmology, Royal Victoria Eye and Ear Hospital, Adelaide Rd, Dublin 2, D02 XK51 Ireland

**Keywords:** Cancer, COVID-19, Uveal melanoma

## Abstract

**Aim:**

We aim to evaluate the impact of the COVID-19 pandemic on ocular oncology in Ireland, comparing uveal melanoma trends in 2019 to 2020.

**Methods:**

Patients included for analysis were those that presented to the ocular oncology service from January 2019 to December 2020 in the Royal Victoria Eye and Ear Hospital in Dublin, who underwent primary treatment for uveal melanoma—proton beam therapy, brachytherapy or enucleation.

**Results:**

Ninety-seven patients presented in 2019 (*n* = 46) and 2020 (*n* = 51) who underwent primary treatment for uveal melanoma. Presentation via the eye casualty department was more common in 2020. Dimensions of choroidal melanomas were increased both in basal diameter and thickness compared to those in 2019. More patients had enucleations in 2020 than in 2019 (21.6% vs 9.3%, respectively) and less had proton beam therapy (6.2% vs 12.4%). More patients had evidence of extra-scleral extension at the time of surgery in 2020 compared to 2019 (4.1%, *n* = 4 versus 0%, respectively). The mean duration of brachytherapy therapy was longer in 2020 (5.3 days ± 35.8) compared to 2019 (4.6 days ± 38.7). Mean time between presentation and primary treatment was 35.6 ± 28.8 days in 2019 and 24.1 ± 20.4 days in 2020.

**Conclusions:**

More advanced disease is suggested by the increased mean basal diameter and tumour thickness, extra-scleral extension and longer duration of brachytherapy. Time from diagnosis to treatment was not delayed in 2020.

## Introduction

The COVID-19 pandemic is an ever-evolving situation and an ongoing challenge to our already stretched healthcare system. The treatment of ocular malignancies is considered non-deferrable and should proceed despite the pandemic. Delays in the treatment of these patients may result in negative survival outcomes. We aim to evaluate the impact of the COVID-19 pandemic on ocular oncology in Ireland, comparing uveal melanoma trends in 2019 to 2020.

## Methods

Data was collected from our local ocular oncology database, retrospective analysis of electronic notes and review of patient charts. Patients included for analysis were those that presented to the ocular oncology service from January 2019 to December 2020 in the Royal Victoria Eye and Ear Hospital in Dublin, who underwent primary treatment for uveal melanoma—proton beam therapy, brachytherapy or enucleation. Information was collected regarding patient demographics (age, gender, smoking status, ethnicity), clinical features (laterality—right or left, site of uveal melanoma, e.g. ciliochoroidal/juxta-papillary/choroidal, basal diameter and thickness, date of presentation, date of primary treatment, primary treatment received, e.g. proton beam, brachytherapy or enucleation, plaque duration and type, presence or absence of extra-scleral extension upon enucleation) as well as chromosomal and histological features, e.g. cell type, disomy/monosomy 3, BAP 1. Excluded for analysis were patients that presented to our service prior to January 2019 or after December 2020, patients with non-uveal ocular melanomas, e.g. conjunctival melanomas, those undergoing conservative/observational or palliative management, those refusing treatment and exenterations for orbital tumours. A diagnosis of uveal melanoma was made based on clinical features and examination findings from a dilated fundus examination and multimodal imaging consisting of colour fundus photography, optical coherence tomography and B-scan ultrasonography. An ocular oncology specialist formulated a treatment plan in consultation with a multidisciplinary team. DNA mutations in the BAP1 gene were evaluated by immunohistochemical staining of formalin-fixed paraffin-embedded sections. Uveal material was tested for loss of chromosome 3 and gain of chromosome 8q gene signatures by selective molecular gene markers using multiplex ligation-dependent probe amplification (MLPA). Statistical analyses were conducted using SAS JMP data analysis software Version 16.1.0. Categorical variables were analysed using *χ*^2^ tests and continuous variables were analysed using the Kruskal–Wallis and Mann–Whitney tests, using a two-tailed significance level of *P* < 0.05.

**Table 1:** Summary of uveal melanoma characteristics and demographics, 2019 and 2020

	**2019**	**2020**
Sex *N* (%)		
Female	15 (32.6)	20 (39.2)
Male	31 (67.4)	31 (60.8)
Total	46 (100)	51 (100)
Age, mean ± SD	61.1 ± 11.1	61.2 ± 12.8
Source of referral *N* (%)		
Casualty	1 (2.2)	7 (13.7)
Diabetic screening	1 (2.2)	0 (0)
Optician	2 (4.3)	2 (4.3)
Ophthalmologist	42 (91.3)	40 (78.4)
Tumour dimension, mean ± SD		
Basal diameter (mm)	11.8 ± 4.0	13.4 ± 3.9
Thickness (mm)	4.8 ± 2.7	6.6 ± 3.5
Primary treatment *N*(%)		
Proton beam therapy	12 (12.4)	6 (6.2)
Brachytherapy	25 (25.8)	24 (24.7)
Enucleation	9 (9.3)	21 (21.6)
Presence of extra-scleral extension	0 (0)	4 (4.1)
Time from presentation to treatment (mean ± SD)		
Overall (days)	35.6 ± 28.8	24.1 ± 20.4

**Fig. 1:**
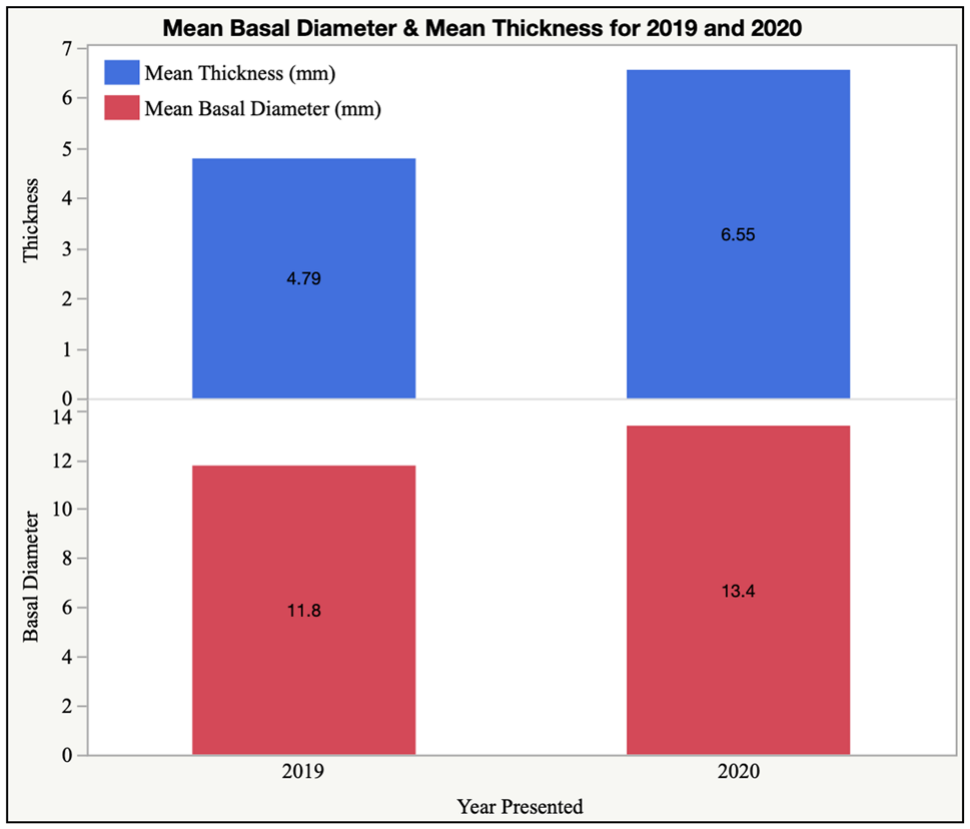
Uveal melanoma: mean basal diameter and tumour thickness (mm) in 2019 and 2020

## Results

Ninety-seven patients presented in 2019 (*n* = 46) and 2020 (*n* = 51) who underwent primary treatment for uveal melanoma: proton beam therapy, brachytherapy or enucleation. More males (64.3%, *n* = 62) than females (35.7%, *n* = 35) presented, both in 2019 to 2020. Mean age of presentation was 61.1 ± 11.1 (range 32.0–82.2) in 2019 and 61.2 ± 12.8 (range 32.4–81.8) in 2020. Patients identified as either Irish (94.8%, *n* = 92), English (3.1%, *n* = 3) or Lithuanian (2.1%, *n* = 2). Referrals came from 20 counties in Ireland, predominantly from County Dublin in both 2019 (*n* = 19, 19.6%) and 2020 (*n* = 14, 14.4%). Most patients were non-smokers (79.4%, *n* = 77), 14% (*n* = 14) were smokers and 6.2% (*n* = 6) identified as ex smokers. There was no significant difference in smoking status between presentations in 2019 and 2020 (*P* = 0.45). The main source of referral to the ocular oncology service was via another ophthalmologist (84.5%, *n* = 82), 91.3% (*n* = 42) in 2019 and 78.4% (*n* = 40) in 2020. The source of referral was more frequently via the eye casualty department in 2020 (13.7%, *n* = 7) compared to 2019 (2.2%, *n* = 1); however, this difference was not statistically significant (*P* = 0.12). Other sources of referral included diabetic screening service (2.2%, *n* = 1 in 2019, 0% in 2020) or the optician (4.3%, *n* = 2 in 2019, 4.3%, *n* = 2 in 2020).

Eyes included in the study were mainly right eyes (56.7%, *n* = 55) versus left eyes (43.3%, *n* = 42). The dimensions of choroidal melanomas on presentation in 2020 were increased both in basal diameter and thickness compared to those in 2019. Mean basal diameter was 11.8 ± 4.0 mm (range 3.0–18.6 mm, *n* = 46) and 13.4 ± 3.9 mm (range 5.2–21.6 mm, *n* = 51) in 2019 and 2020, respectively (*P* < 0.05). Mean tumour thickness on presentation was 4.8 ± 2.7 (range 1.2–13.1, *n* = 46) and 6.6 ± 3.5 (range 1.1–16.0 mm, *n* = 51) in 2019 and 2020, respectively (*P* < 0.01) (Fig. 1). Males presented with increased uveal tumour dimensions compared to their female counterparts in both 2019 and 2020; however, this result was not statistically significant (*P* = 0.34). Mean tumour dimensions increased in females, from 11.0 ± 4.6 mm basal diameter and 4.6 ± 3.2 mm thickness in 2019, to 12.5 ± 4.0 mm basal diameter and 5.4 ± 2.8 mm thickness in 2020. Mean tumour dimensions also increased in males, from 12.1 ± 3.7 mm basal diameter and 4.9 ± 2.5 mm thickness in 2019 to 13.9 ± 3.7 mm basal diameter and 7.3 ± 3.7 mm thickness in 2020.

Of those receiving genetic analysis (*n* = 51), a third of patients had monosomy 3 (33.3%, *n* = 17), 41.2% (*n* = 21) had chromosomal 8 alterations. BRCA1-associated protein 1 (BAP1) gene mutations were identified in 14.3% (*n* = 6) of those analysed (*n* = 42). Cell analysis was performed in fifty eyes, showing spindle predominance in 40% (*n* = 20) mixed in 24% (*n* = 12) and epithelioid in 12% (*n* = 6), with insufficient material for analysis in another 24% (*n* = 12). There was no significant difference in the genetics nor the histology of uveal tumours from 2019 to 2020.

Half our patients underwent brachytherapy as their primary treatment (50.5%, *n* = 49), 30.9% (*n* = 30) had enucleation and 18.6% (*n* = 18) had proton beam therapy. Significantly more patients had enucleations as their primary treatment in 2020 than 2019 (21.6% vs 9.3%, respectively), and less had proton beam therapy as their primary treatment (6.2% vs 12.4%) (*P* < 0.05). Overall, 94.8% of primary enucleations had no extra-scleral extension noted at the time of surgery in 2019 and 2020 (*n* = 30). More patients had evidence of extra-scleral extension at the time of surgery in 2020 compared to 2019 (4.1%, *n* = 4 versus 0%, respectively) (*P* < 0.05). Of those that underwent brachytherapy, 81.3% (*n* = 39) had a Ru-106 (Ruthenium-106) plaque and 18.8% (*n* = 18) received a I-125 (Iodine-125) plaque. The mean duration of brachytherapy therapy was longer in 2020 (5.3 days ± 35.8) compared to 2019 (4.6 days ± 38.7); however, this difference was not significant (*P* < 0.112). Time from diagnosis to treatment was not increased in 2020. The timeline between presentation to the ocular oncology service and primary treatment was 35.6 ± 28.8 days in 2019 and 24.1 ± 20.4 days in 2020 (*P* < 0.05)(Table 1).

## Discussion

The COVID-19 (SARS-CoV-2 2019) outbreak resulted in an unprecedented disruption to healthcare as we know it, an attempt to limit the exposure of patients to the virus contagion, without compromising healthcare. The pandemic led to reprioritisation of non-emergency services, including the diagnostics and elective specialist surgery and redeployment of staff. Delays in diagnosis and treatment of cancer can have a negative impact on patients’ survival [1]. Like other cancer services, the Royal Victoria Eye and Ear Hospital tertiary referral oncology service remained fully operative. This article describes the presentation and management of patients with uveal melanomas in Ireland in 2019 and 2020, to determine if this pandemic led to more advanced presentations or delays to treatment.

Advice on staying at home, restricted travel and fear of contracting COVID-19 has likely resulted in delayed presentations and more advanced disease on initial presentation. While some patients present with symptoms, other are diagnosed by dilated ocular examinations for other reasons; the cessation of scheduled optometry and ophthalmology appointments may have contributed to more patients presenting via the eye casualty department in 2020 compared to 2019 (7.3% vs 1%). This suggests they presented with symptoms rather than an incidental finding. The American Association of Ophthalmic Oncologists and Pathologists (AAOOP) issued recommendations that the treatment of ocular malignancies proceed during the COVID-19 pandemic [2]. Reassuringly, the actual volume of patients diagnosed with uveal melanoma requiring primary treatment (proton beam therapy, enucleation or brachytherapy) was not notably different between 2019 and 2020 (*n* = 46 vs *n* = 51), and in keeping with previous reports regarding incidence of uveal melanoma in Ireland from 2010 to 2015 (annual average 45 cases per year, range 34–61) [3].

The pandemic impact is not unique to Ireland. A report from the Netherlands notes a 27% decrease in the rate of new cancer diagnosis during the COVID-19 pandemic and a 6-month period of restrictions in Victoria, Australia, saw a 10% reduction in cancers diagnosed [4, 5]. The UK confirms a reduction in referral numbers by 42% for eye cancer and estimates 120 fewer uveal melanoma cases compared to previous years [6]. Increasing presentations of more advanced disease is a potential consequence of the COVID-19 pandemic. More advanced disease presentations are suggested by the increased mean basal diameter and mean thickness of uveal melanomas presenting in 2020 compared to not just in 2019, but also compared to the published data from 2010 to 2015 in Ireland [3]. Increased thickness negatively impacts distant metastases-free survival rates [3]. We note the mean tumour thickness as being greater in males than in females, a trend previously outlined by Bailey et al., with a male:female incidence ratio of 1.35 (2010–2015) [3]. Similar trends were extracted from the UK Liverpool Ocular Oncology Centre (LOOC) database looking at 3380 patients from 1993 to 2010; the median largest basal tumour diameter was 12.2 mm in men and 11.9 mm in women (*P* = 0.001), and tumour thickness had a median of 4.4 mm and 3.8 mm in men and women, respectively (*P* = 0.015) [6].

Chromosome 3 and 8 abnormalities and germ-line BAP1 mutations correlate with traditional factors of poor prognosis, such as larger tumour diameters [7]. There was no significant difference in the genetics or histology of uveal tumours comparing 2019 to 2020; not a contributory factor to explain the larger presenting tumours, nor a reason to influence treatment modalities. Higher percentages of patients underwent enucleation and higher percentages of enucleations had extra-scleral extension noted during surgery in 2020 compared to 2019 (4.1% vs 0%, *P* < 0.05), suggesting more advanced disease. Proton beam therapy was performed less frequently during 2020 compared to 2019 (6.2% vs 12.4%). This is a similar trend to a multi-centre retrospective review of adult eye oncology centres in the UK during the COVID-19 pandemic, in which more patients underwent enucleation during the 4-month period between March-June 2020 compared to the mean of previous 2 years (27.7% vs 22.8%) (*P* = 0.229) and proton beam therapy was performed less frequently (17.5%) during the lockdown period compared to the mean (17.5% vs 29.2%) (*P* = 0.011) from the same periods in 2018 and 2019 [6].

The number of patients receiving plaque brachytherapy (Ruthenium-106 or Iodine-125) was similar in 2019 as in 2020 (*n* = 25 vs *n* = 24, respectively). Brachytherapy requires hospital admission for up to a week and includes two surgical procedures and general anaesthesia; insertion and subsequent removal of the plaque once the radiation dose have been delivered. This globe-saving treatment option was therefore not avoided in an attempt to reduce hospital contact. In fact, the brachytherapy plaque duration and resultant inpatient stay was actually longer in 2020 compared to 2019 (5.3 vs 4.6 days). This again suggests more advanced tumours, requiring a higher apex brachytherapy dose. Importantly, time from diagnosis to treatment was not delayed in 2020 compared to 2019. Difference between presentation to the oncology service and obtaining primary treatment was 35.6 ± 28.8 days in 2019 and 24.1 ± 20.4 days in 2020 (*P* < 0.05). We postulate this reduced time being due to the prioritisation of surgical oncology cases, especially those presenting at advanced stages. As the pandemic worsens, we may return to prioritising surgical oncology cases, a precedent set by the American College of Surgeons [8]. Patients need to be evaluated on a case-by-case basis and available resources may vary internationally. Telemedicine and virtual multidisciplinary discussions can be utilised; we can review notes and imaging prior to the consultation to shorten potential exposure. At this point in the pandemic, there is no decision to alter treatment algorithms to favour enucleation over globe salvage [2]. We may take guidance by the practice patterns of others, such as those available from the Collaborative Ocular Oncology Group, recommending that newly diagnosed patients continue to be seen [9].

## Conclusions

The Irish government COVID-19 National Action Plan identified the continued delivery of cancer care as a priority [10]. Reassuringly, the same caseload of patients presented for primary treatment of uveal melanoma in 2019 as in 2020. Patients presented with more advanced disease in 2020: uveal melanoma dimensions on presentation in 2020 were increased both in basal diameter and thickness compared to 2019 (*P* > 0.05). More enucleations were performed during the COVID-19 period compared to the previous year and higher percentages of patients who underwent enucleation had evidence of extra-scleral extension at the time of surgery (*P* > 0.05). Time from diagnosis to primary treatment was not increased from 2019 to 2020. Risk of COVID-19 transmission to patients must be minimised but also their burden of disease, while balancing the availability of local resources. These decisions are not simple. The ocular oncology service will continue to face challenges, we may yet see increasing numbers presenting with more advanced disease post lockdown. The greatest threat to resilience in the cancer services in the short-term remains increased community transmission of COVID-19. It is important that we continue to promote the message that our cancer diagnostic and treatment services are open.

## Data Availability

The datasets generated during the current study are available from the corresponding author on reasonable request.
